# The role of spatially varying loadings in dynamic spatial factor models for modeling the opioid syndemic

**DOI:** 10.1007/s10742-025-00356-7

**Published:** 2025-09-16

**Authors:** Eva Murphy, David Kline, Staci A. Hepler

**Affiliations:** 1 Wake Forest University, Winston-Salem, USA; 2 Wake Forest University School of Medicine, Winston-Salem, USA

**Keywords:** Spatial Loadings, Bayesian Factor Models, Factor Covariance, Opioid Epidemic

## Abstract

Understanding the interactions and spatio-temporal variations of public health outcomes is crucial for gaining insight into interrelated epidemics across different locations and time periods. Dynamic spatial factor models provide a flexible framework for capturing shared variability among multiple outcomes through a latent factor and its corresponding loadings. A common assumption in these models is that factor loadings are spatially constant, implying uniform relationships between outcomes across the study region. However, this assumption may overlook important regional differences in how outcomes relate to the underlying latent factor. In this study, we derive the covariance structure of the outcome vector to highlight how spatially varying versus constant loadings influence the overall correlation structure. We find that when loadings vary across space, the spatial covariance of the outcomes is shaped by both the spatial covariance of the loadings and the latent factors. In contrast, when loadings are spatially constant, the spatial covariance of the outcomes is determined primarily by the latent factors, leading to uniform variation across the spatial domain. To assess these differences in practice, we apply a Bayesian hierarchical spatial dynamic factor model to analyze the opioid syndemic in North Carolina. Our results suggest that incorporating spatially varying loadings provides a more detailed, county-specific understanding of the epidemic. This added flexibility enables a localized interpretation of opioid-related interactions and offers insights that could inform targeted public health interventions.

## 1 Introduction

Factor analysis provides a flexible statistical framework for modeling correlated outcomes by identifying shared variation and interconnected dynamics through the use of latent factors. Factor models have been widely used in public health to understand complex relationships between epidemics or other interconnected health outcomes. For example, they have been used to jointly explore multiple diet-related cancer mortality rates (Tzala and Best 2008); examine interactions between environmental variables such as particulate matter $\left(PM_{10}\right)$ and nitrogen oxide (Ippoliti et al. 2012); investigate the effects of speciated components of particulate matter $\left(PM_{2.5}\right)$ on cleft birth defects (Kaufeld et al. 2017); analyze interactions between opioid-related outcomes (Hepler et al. 2019; Kline et al. 2023; Murphy et al. 2025); and study disease-specific death rates alongside socio-economic and behavioral indicators to identify common spatial patterns in morbidity determinants and assess their variations over time (Leorato and Mezzetti 2021).

Factor models assume that each observed outcome is a linear combination of latent factors plus measurement error. Letting $\text{Y}={\left(y_{1},\ldots ,y_{k}\right)}^{T}$ denote the k×1 vector of correlated outcomes, $\text{F}={\left(F_{1},\ldots ,F_{m}\right)}^{T}$ the vector of factors, and assuming no intercept, their relationship can be expressed as:

(1) $$Y=\Gamma F+\epsilon ,\epsilon \sim N_{k}\left(0,\Sigma \right),\;F\sim N_{m}\left(0,\Phi \right),$$

where Γ is a k×m matrix of factor loadings, ϵ the measurement error, $\Sigma =\text{diag}\left(\sigma_{1}^{2},\ldots ,\sigma_{k}^{2}\right),\Phi$ is an m×m matrix that captures the covariance structure of the latent factors, and $N_{k}(\cdot ,\cdot )$ denotes the k-dimensional multivariate normal distribution with the specified mean vector and covariance matrix. The role of the factor loadings, Γ, is to quantify the relationship between the factor and the outcomes. Higher loadings suggest a stronger relationship, while values near zero imply a weaker relationship. Furthermore, the sign of the loading reveals whether the latent factor and observed variable are positively or negatively correlated.

Wang and Wall (2003) extend the common factor model to a generalized spatial factor model, enabling the analysis of a broader range of observed data from the exponential family, including Poisson and binomial data in space. In this framework, eq. (1) takes the form:

(2) $$Y_{i}=\Gamma F_{i}+\varepsilon_{i},$$

where the index i represents a location, i=1,…,N. Their work opens the door to more flexible modeling approaches for public health data, allowing researchers to quantify the variation within correlated health outcomes across space. Lopes et al. (2008) further develop the spatial factor model by incorporating temporal dynamics, allowing outcomes to be analyzed over time. In their approach, spatial dependence is modeled through the factor loadings, while temporal dependence is captured by the latent factors. This added flexibility enhances the model’s ability to capture complex spatio-temporal relationships, generalizing previous methods.

It follows from (1), that the distribution of the outcome vector after marginalizing over the latent factor is $Y|\Gamma \sim N_{k}\left(0,\Gamma \Phi \Gamma^{T}+\Sigma \right)$. The decomposition of the variance-covariance structure of Y, conditional on Γ, shows that the correlation among outcomes is explained by the factor loadings, the covariance of the latent factors, and the measurement error variances. This highlights the ability of factor models to identify patterns in the data and effectively capture interactions between outcomes. However, this decomposition is not unique making Γ and Φ unidentifiable. To address this, various constraints are applied to the model parameters, with a common approach being the use of a lower triangular structure with positive (or unit) diagonal elements for the loadings matrix (Geweke and Zhou 1996; Frühwirth-Schnatter and Lopes 2018). Murphy et al. (2025) propose an alternative solution that relaxes the strict lower triangular constraint by applying an LQ decomposition to the unidentifiable loadings matrix. This decomposition results in a lower triangular matrix L with positive diagonal elements and an orthogonal matrix Q. By enforcing strictly positive diagonal elements in L, the resulting lower triangular matrix aligns with previously mentioned loadings constraints. Assuming in addition uncorrelated factors with unit variance ensures unique solutions to (1).

Our work is motivated by the study of the opioid syndemic. That is, the synergy of interrelated conditions including opioid use disorder, overdoses (fatal and non-fatal), and the transmission of human immunodeficiency virus (HIV) and hepatitis C virus (HCV) (Perlman and Jordan 2018; Singer et al. 2017). Spatial and spatio-temporal factor models provide a valuable framework for modeling syndemics and the interactions between opioid-related outcomes. These interactions, and implicitly the syndemic, are expected to vary across locations, as counties experience the syndemic in different ways. Therefore, it is important to account for how the factor influences the outcomes at the location-specific level. Since the loadings quantify the factor’s influence on the outcomes, the structure of the loadings becomes important. Modeling the loadings as spatially varying allows us to capture this location-specific variability, providing insights into how the same latent factor might affect outcomes differently across areas. If the loadings are assumed to be constant across all locations, we implicitly assume that the factor influences outcomes uniformly, potentially overlooking important regional differences. To address this, our goal is to investigate whether spatially modeling the loadings provides a more nuanced approach to understanding the opioid syndemic.

We present two sides of our analysis that directly address our goal. Building on the key idea that the factor and the loadings capture sources of variability within and between the outcomes, we first analyze the covariance structure of the outcomes to understand how the spatially varying loadings influence both the within-outcome and between-outcome covariance. Second, we analyze real-world data on six opioid-related outcomes — illicit opioid overdose deaths, emergency department visits for drug overdose, opioid use disorder treatment counts, buprenorphine prescriptions, and new diagnoses of HCV and HIV — to explore the practical implications of using spatially varying versus spatially constant loadings.

The remainder of the paper is structured as follows: in sect. 2 we derive the spatial covariance structure of the outcome vector after marginalizing over both the latent factor and loadings. In sect. 3 we present the modeling framework and a comparison of results obtained using spatially varying loadings versus non-spatially varying loadings in an application setting. We conclude with a discussion in sect. 4.

## 2 Factor model covariance

The assumption of spatially constant loadings provides an overall summary, such as a state-level average, of the relationship between observed outcomes and the latent factor. However, regions are not uniform, and differences in geographic, demographic, or economic conditions may lead to varying patterns in the underlying relationships across locations. In this section, we derive the mathematical formula for the covariance-variance structure of the outcomes assumed by the prior model specifications to better understand how the factor and loadings contribute to the variation within and between outcomes. This allows us to compare the structure when loadings are spatially varying versus when they remain constant, offering insights into how spatial variability influences these interactions.

Assume, without loss of generality, only three outcomes. That is, at location i we have $Y_{i}=\left(Y_{i}^{\left(1\right)},Y_{i}^{\left(2\right)},Y_{i}^{\left(3\right)}\right)$ for i=1,…,N, and the outcomes are governed by a single factor, $f_{i}$. Specifically, we assume the following spatial factor model:

(3) $$Y_{i}=\Gamma_{i}f_{i}+\varepsilon_{i},$$

where $f_{i}$ is the factor corresponding to location $i,\Gamma_{i}$ is now a 3 × 1 vector of loadings, and $\varepsilon_{i}=\left(\varepsilon_{i}^{\left(1\right)},\varepsilon_{i}^{\left(2\right)},\varepsilon_{i}^{\left(3\right)}\right)$ are the outcome specific error terms, such that $\varepsilon_{i}^{\left(k\right)}\overset{\text{ind}}{\sim }N\left(0,\sigma_{\left(k\right)}^{2}\right),k=1,2,3$, and are independent across space and outcome. To ensure identifiability, we fix the loading of the first outcome to 1 at all locations, so that $\Gamma_{i}={\left(1,\gamma_{i}^{\left(2\right)},\gamma_{i}^{\left(3\right)}\right)}^{T},i=1,\ldots ,N$. For this illustration, we further assume *a priori* that the latent factors, $f_{i}$, have a spatial model such that the resulting joint distribution is of the form $F={\left(f_{1},f_{2},\ldots ,f_{N}\right)}^{T}\sim N_{N}\left(0,\Sigma^{F}\right)$, where $\Sigma^{F}$ represents the spatial covariance structure of the factor vector. Similarly, assume the loadings have a spatial model such that $\gamma^{\left(k\right)}=\left(\gamma_{1}^{\left(k\right)},\ldots ,\gamma_{N}^{\left(k\right)}\right)\sim N\left(\mu^{\left(k\right)},\Sigma^{\Gamma^{\left(k\right)}}\right)$, where $\mu^{\left(k\right)}=\left(\mu_{1}^{\left(k\right)},\ldots ,\mu_{N}^{\left(k\right)}\right)$ denotes the mean vector and $\Sigma^{\Gamma^{\left(k\right)}}$ denotes the spatial covariance matrix pertaining to outcome k,k=2,3. Finally, assume that $f_{i},\Gamma_{i}$ and $\varepsilon_{i}$ are independent of one another for all i=1,…,N.

Define $Y={\left(Y_{1:N}^{\left(1\right)},Y_{1:N}^{\left(2\right)},Y_{1:N}^{\left(3\right)}\right)}^{T}$ as the vector of outcomes across all locations, where $Y_{1:N}^{\left(k\right)}$ represents the vector of the $k^{\text{th}}$ outcome (k=1,2,3) for all locations. With this, we can express (3) in matrix form as follows:

$$Y=\left(\begin{array}{c} Y_{1}^{\left(1\right)}\\ Y_{2}^{\left(1\right)}\\ \vdots \\ Y_{N}^{\left(1\right)}\\ Y_{1}^{\left(2\right)}\\ Y_{2}^{\left(2\right)}\\ \vdots \\ Y_{N}^{\left(2\right)}\\ Y_{1}^{\left(3\right)}\\ Y_{2}^{\left(3\right)}\\ \vdots \\ Y_{N}^{\left(3\right)} \end{array}\right)=\left(\begin{array}{cccc} 1 & 0 & \ldots & 0\\ 0 & 1 & \ldots & 0\\ \vdots & \vdots & \ddots & \vdots \\ 0 & 0 & \ldots & 1\\ \gamma_{1}^{\left(2\right)} & 0 & \ldots & 0\\ 0 & \gamma_{2}^{\left(2\right)} & \ldots & 0\\ \vdots & \vdots & \ddots & \vdots \\ 0 & 0 & \ldots & \gamma_{N}^{\left(2\right)}\\ \gamma_{1}^{\left(3\right)} & 0 & \ldots & 0\\ 0 & \gamma_{2}^{\left(3\right)} & \ldots & 0\\ \vdots & \vdots & \ddots & \vdots \\ 0 & 0 & \ldots & \gamma_{N}^{\left(3\right)} \end{array}\right)\cdot \left(\begin{array}{c} f_{1}\\ f_{2}\\ \vdots \\ f_{N} \end{array}\right)+\left(\begin{array}{c} \varepsilon_{1}^{\left(1\right)}\\ \varepsilon_{2}^{\left(1\right)}\\ \vdots \\ \varepsilon_{N}^{\left(1\right)}\\ \varepsilon_{1}^{\left(2\right)}\\ \varepsilon_{2}^{\left(2\right)}\\ \vdots \\ \varepsilon_{N}^{\left(2\right)}\\ \varepsilon_{1}^{\left(3\right)}\\ \varepsilon_{2}^{\left(3\right)}\\ \vdots \\ \varepsilon_{N}^{\left(3\right)} \end{array}\right).$$

It follows that the cross-covariance matrix can be written in block form as

(4) $$\text{Cov}\left(Y\right)=\left(\begin{array}{ccc} \text{Cov}\left(Y_{1:N}^{\left(1\right)}\right) & \text{Cov}\left(Y_{1:N}^{\left(1\right)},Y_{1:N}^{\left(2\right)}\right) & \text{Cov}\left(Y_{1:N}^{\left(1\right)},Y_{1:N}^{\left(3\right)}\right)\\ \text{Cov}\left(Y_{1:N}^{\left(2\right)},Y_{1:N}^{\left(1\right)}\right) & \text{Cov}\left(Y_{1:N}^{\left(2\right)}\right) & \text{Cov}\left(Y_{1:N}^{\left(2\right)},Y_{1:N}^{\left(3\right)}\right)\\ \text{Cov}\left(Y_{1:N}^{\left(3\right)},Y_{1:N}^{\left(1\right)}\right) & \text{Cov}\left(Y_{1:N}^{\left(3\right)},Y_{1:N}^{\left(2\right)}\right) & \text{Cov}\left(Y_{1:N}^{\left(3\right)}\right) \end{array}\right),$$

where each element represents a covariance block matrix. Specifically, the diagonal blocks correspond to the covariance matrices of the $k^{\text{th}}$ outcome for k=1,2,3, while the off-diagonal blocks represent the cross-covariance between pairs of outcomes. In what follows, we explicitly express the (i,j) element of each block to provide a clearer understanding of its structure. We begin with $\text{Cov}\left(Y_{1:N}^{\left(1\right)}\right)$, whose (i,j) entry is given by:

(5) $$\text{Cov}\left(Y_{i}^{\left(1\right)},Y_{j}^{\left(1\right)}\right)=E\left[\left(Y_{i}^{\left(1\right)}-E\left[Y_{i}^{\left(1\right)}\right]\right)\left(Y_{j}^{\left(1\right)}-E\left[Y_{j}^{\left(1\right)}\right]\right)\right]\;\begin{array}{c} =E\left[\left(Y_{i}^{\left(1\right)}\right)\left(Y_{j}^{\left(1\right)}\right)\right] \end{array}$$

(6) $$=E\left[\left(1\cdot f_{i}+\varepsilon_{i}^{\left(1\right)}\right)\left(1\cdot f_{j}+\varepsilon_{j}^{\left(1\right)}\right)\right]\;=E\left[\left(f_{i}\cdot f_{j}+f_{i}\cdot \varepsilon_{j}^{\left(1\right)}+\varepsilon_{i}^{\left(1\right)}\cdot f_{j}+\varepsilon_{i}^{\left(1\right)}\cdot \varepsilon_{j}^{\left(1\right)}\right)\right]\;=E\left[f_{i}\cdot f_{j}\right]+E\left[\left(\varepsilon_{i}^{\left(1\right)}\cdot \varepsilon_{j}^{\left(1\right)}\right]\right.$$

(7) $$=\Sigma_{\text{ij}}^{F}+\delta_{\text{ij}}\cdot \sigma_{\left(1\right)}^{2},$$

where $\Sigma_{\text{ij}}^{F}$ denotes the (i,j) entry of the spatial covariance matrix of F and $\delta_{\text{ij}}$ denotes the Kronecker delta, which is 1 if i=j and 0 otherwise. In (5) we used that $E\left[Y_{i}^{\left(1\right)}\right]=E\left[Y_{j}^{\left(1\right)}\right]=0$, in (6) we used that $f_{i}$ and $f_{j}$ are independent from $\varepsilon_{i}^{\left(1\right)}$ and $\varepsilon_{j}^{\left(1\right)}$, respectively, and in (7) that $E\left[f_{i}f_{j}\right]=\text{Cov}\left(f_{i},f_{j}\right)+E\left[f_{i}\right]E\left[f_{j}\right]=\Sigma_{\text{ij}}^{F}+0=\Sigma_{\text{ij}}^{F}$. Similarly, the (i,j) element of $\text{Cov}\left(Y_{1:N}^{\left(k\right)}\right),k=2,3$, is defined as follows:

(8) $$\begin{array}{ll} \text{Cov}\left(Y_{i}^{\left(k\right)},Y_{j}^{\left(k\right)}\right) & =E\left[\left(Y_{i}^{\left(k\right)}-E\left[Y_{i}^{\left(k\right)}\right]\right)\left(Y_{j}^{\left(k\right)}-E\left[Y_{j}^{\left(k\right)}\right]\right)\right]\\ & =E\left[\left(\gamma_{i}^{\left(k\right)}\cdot f_{i}+\varepsilon_{i}^{\left(k\right)}\right)\left(\gamma_{j}^{\left(k\right)}\cdot f_{j}+\varepsilon_{j}^{\left(k\right)}\right)\right]\\ & =E\left[\gamma_{i}^{\left(k\right)}\cdot \gamma_{j}^{\left(k\right)}\cdot f_{i}\cdot f_{j}\right]+E\left[\varepsilon_{i}^{\left(k\right)}\cdot \varepsilon_{j}^{\left(k\right)}\right]\\ & =E\left[\gamma_{i}^{\left(k\right)}\cdot \gamma_{j}^{\left(k\right)}\right]E\left[f_{i}\cdot f_{j}\right]+\delta_{\text{ij}}\cdot \sigma_{\left(k\right)}^{2}\\ & =\left(\Sigma_{\text{ij}}^{\Gamma^{\left(k\right)}}+\mu_{i}^{\left(k\right)}\mu_{j}^{\left(k\right)}\right)\Sigma_{\text{ij}}^{F}+\delta_{\text{ij}}\cdot \sigma_{\left(k\right)}^{2}, \end{array}$$

where $\Sigma_{\text{ij}}^{\Gamma^{\left(k\right)}}$ denotes (i,j) entry of the spatial covariance that corresponds to the loadings associated with outcome k between locations i and j. In (8) we used that $E\left[\gamma_{i}^{\left(k\right)}\gamma_{j}^{\left(k\right)}\right]=\text{Cov}\left(\gamma_{i}^{\left(k\right)},\gamma_{j}^{\left(k\right)}\right)+E\left[\gamma_{i}^{\left(k\right)}\right]E\left[\gamma_{j}^{\left(k\right)}\right]=\Sigma_{\text{ij}}^{\Gamma^{\left(k\right)}}+\mu_{i}^{\left(k\right)}\mu_{j}^{\left(k\right)}$.

The (i,j) element of $\text{Cov}\left(Y_{1:N}^{\left(1\right)},Y_{1:N}^{\left(k\right)}\right)$, for k=2,3, is

(9) $$\begin{array}{ll} \text{Cov}\left(Y_{i}^{\left(1\right)},Y_{j}^{\left(k\right)}\right) & =E\left[\left(Y_{i}^{\left(1\right)}-E\left[Y_{i}^{\left(1\right)}\right]\right)\left(Y_{j}^{\left(k\right)}-E\left[Y_{j}^{\left(k\right)}\right]\right)\right]\\ & =E\left[\left(1\cdot f_{i}+\varepsilon_{i}^{\left(1\right)}\right)\left(\gamma_{j}^{\left(k\right)}\cdot f_{j}+\varepsilon_{j}^{\left(k\right)}\right)\right]\\ & =E\left[1\cdot \gamma_{j}^{\left(k\right)}\cdot f_{i}\cdot f_{j}\right]+E\left[\varepsilon_{i}^{\left(1\right)}\cdot \varepsilon_{j}^{\left(k\right)}\right]\\ & =E\left[1\cdot \gamma_{j}^{\left(k\right)}\right]E\left[f_{i}\cdot f_{j}\right]+0\\ & =\mu_{j}^{\left(k\right)}\cdot \Sigma_{\text{ij}}^{F}. \end{array}$$

Finally, the (i,j) element of $\text{Cov}\left(Y_{1:N}^{\left(2\right)},Y_{1:N}^{\left(3\right)}\right)$ is

(10) $$\begin{array}{ll} \text{Cov}\left(Y_{i}^{\left(2\right)},Y_{j}^{\left(3\right)}\right) & =E\left[\left(Y_{i}^{\left(2\right)}-E\left[Y_{i}^{\left(2\right)}\right]\right)\left(Y_{j}^{\left(3\right)}-E\left[Y_{j}^{\left(3\right)}\right]\right)\right]\\ & ==E\left[\left(\gamma_{i}^{\left(2\right)}\cdot f_{i}+\varepsilon_{i}^{\left(2\right)}\right)\left(\gamma_{j}^{\left(3\right)}\cdot f_{j}+\varepsilon_{j}^{\left(3\right)}\right)\right]\\ & =E\left[\gamma_{i}^{\left(2\right)}\cdot \gamma_{j}^{\left(3\right)}\cdot f_{i}\cdot f_{j}\right]+E\left[\varepsilon_{i}^{\left(2\right)}\cdot \varepsilon_{j}^{\left(3\right)}\right]\\ & =E\left[\gamma_{i}^{\left(2\right)}\cdot \gamma_{j}^{\left(3\right)}\right]E\left[f_{i}\cdot f_{j}\right]+0\\ & =\mu_{i}^{\left(2\right)}\mu_{j}^{\left(3\right)}\cdot \Sigma_{\text{ij}}^{F}, \end{array}$$

where we used that $E\left[\gamma_{i}^{\left(2\right)}\gamma_{j}^{\left(3\right)}\right]=\text{Cov}\left(\gamma_{i}^{\left(2\right)},\gamma_{j}^{\left(3\right)}\right)+E\left[\gamma_{i}^{\left(2\right)}\right]E\left[\gamma_{j}^{\left(3\right)}\right]=0+\mu_{i}^{\left(2\right)}\mu_{j}^{\left(3\right)}$.

Using (7)–(10), the covariance in (4) can be expressed as follows:

(11) $$\text{Cov}\left(Y\right)=\left(\begin{array}{ccc} \Sigma^{F}+\Sigma_{\varepsilon }^{\left(1\right)} & \mu^{\left(2\right)}1^{T}⊙\Sigma^{F} & \mu^{\left(3\right)}1^{T}⊙\Sigma^{F}\\ \mu^{\left(2\right)}1^{T}⊙\Sigma^{F} & \left(\Sigma^{\Gamma^{\left(2\right)}}+\mu^{\left(2\right)}{\left(\mu^{\left(2\right)}\right)}^{T}\right)⊙\Sigma^{F}+\Sigma_{\varepsilon }^{\left(2\right)} & \mu^{\left(2\right)}{\left(\mu^{\left(3\right)}\right)}^{T}⊙\Sigma^{F}\\ \mu^{\left(3\right)}1^{T}⊙\Sigma^{F} & \mu^{\left(3\right)}{\left(\mu^{\left(2\right)}\right)}^{T}⊙\Sigma^{F} & \left(\Sigma^{\Gamma^{\left(3\right)}}+\mu^{\left(3\right)}{\left(\mu^{\left(3\right)}\right)}^{T}\right)⊙\Sigma^{F}+\Sigma_{\varepsilon }^{\left(3\right)} \end{array}\right),$$

where ⊙ represents the element-wise multiplication, 1 is an N×1 vector of 1s, $\Sigma_{\varepsilon }^{\left(k\right)}$ is a diagonal matrix, with the diagonal elements equal to $\sigma_{\left(k\right)}^{2},k=1,2,3$. The full covariance matrix, Cov(Y), consists of block matrices, where each diagonal block represents the covariance of one outcome across all locations, and off-diagonal block represents the covariance structure between two outcomes and across all locations.

Equation (11) shows that the spatial covariance of the $k^{\text{th}}$ outcome consists of the spatial variability of the factors for k=1, and for k=2,3, it also includes the spatial variability of the loadings, in addition to the outcome-specific correlation. This decomposition highlights that the spatial variation of a non-reference outcome, where the loadings are not fixed at 1 for identifiability, depends on location-specific variation from the loadings, the factor, and the outcome-specific correlation. A similar conclusion applies to the off-diagonal block matrices: the cross-covariance between two outcomes depends on the spatial variability of the factors and the mean of the loadings at the respective locations.

We close this section by comparing the covariance structure in (11) with the scenario where the loadings do not vary spatially. As mentioned above, this means that the loadings remain constant across all locations, removing the dependence on the index i. Specifically, the constant loadings for the three outcomes are denoted as $\gamma^{\left(1\right)},\gamma^{\left(2\right)}$ and $\gamma^{\left(3\right)}$, respectively. As with the spatially varying loadings, we fix $\gamma^{\left(1\right)}$ to equal 1 for identifiability. Assume $\gamma^{\left(k\right)}\sim N\left(\mu^{\left(k\right)},\tau_{\gamma^{\left(k\right)}}^{2}\right),k=2,3$. In this case, eqs. (7)–(10) become

$$\text{Cov}\left(Y_{i}^{\left(1\right)},Y_{j}^{\left(1\right)}\right)=\Sigma_{\text{ij}}^{F}+\delta_{\text{ij}}\cdot \sigma_{\left(1\right)}^{2},$$

$$\text{Cov}\left(Y_{i}^{\left(k\right)},Y_{j}^{\left(k\right)}\right)=\left(\tau_{\gamma (k)}^{2}+{\left(\mu^{\left(k\right)}\right)}^{2}\right)\Sigma_{\text{ij}}^{F}+\delta_{\text{ij}}\cdot \sigma_{\left(k\right)}^{2},\;k=2,3$$

$$\text{Cov}\left(Y_{i}^{\left(1\right)},Y_{j}^{\left(k\right)}\right)=\mu^{\left(k\right)}\cdot \Sigma_{\text{ij}}^{F},\;k=2,3,$$

$$\text{Cov}\left(Y_{i}^{\left(2\right)},Y_{j}^{\left(3\right)}\right)=\mu^{\left(2\right)}\mu^{\left(3\right)}\Sigma_{\text{ij}}^{F}.$$

The above implies that the covariance matrix of the observed outcomes vector when loadings are spatially constant can be written as follows:

(12) $$\text{Cov}(Y)=\left(\begin{array}{ccc} \Sigma^{F}+\Sigma_{\varepsilon }^{\left(1\right)} & \mu^{\left(2\right)}\Sigma^{F} & \mu^{\left(3\right)}\Sigma^{F}\\ \mu^{\left(2\right)}\Sigma^{F} & \left(\tau_{\gamma^{\left(2\right)}}^{2}+{\left(\mu^{\left(2\right)}\right)}^{2}\right)\cdot \Sigma^{F}+\Sigma_{\varepsilon }^{\left(2\right)} & \mu^{\left(2\right)}\mu^{\left(3\right)}\Sigma^{F}\\ \mu^{\left(3\right)}\Sigma^{F} & \mu^{\left(2\right)}\mu^{\left(3\right)}\Sigma^{F} & \left(\tau_{\gamma^{\left(3\right)}}^{2}+{\left(\mu^{\left(3\right)}\right)}^{2}\right)\cdot \Sigma^{F}+\Sigma_{\varepsilon }^{\left(3\right)} \end{array}\right)$$

where $\Sigma^{F}$ and $\Sigma_{\varepsilon }^{\left(k\right)}(k=1,2,3)$ are as in (11). Comparing eqs. (12) to (11), we observe that within each block, spatial heterogeneity in the covariance is governed solely by the spatial variability in the factor covariance. This implies that the covariance between pairs of outcomes is proportional to the factor covariance between outcomes for every location.

## 3 Modeling the opioid syndemic in North Carolina

In this section, we apply the theoretical framework introduced earlier to real-world data. We first outline the modeling framework used to analyze the interactions among six opioid-related outcomes through a Bayesian spatial dynamic factor model. This approach allows us to capture the spatial and temporal dynamics of the opioid syndemic. We then present the results, highlighting the additional insights that spatially varying loadings provide compared to spatially constant loadings in understanding location differences in the syndemic.

### 3.1 Modeling framework

We adopt the framework proposed by Hepler et al. (2019) to analyze the opioid syndemic in North Carolina (NC), focusing on the synergy between six opioid related outcomes: illicit opioid overdose deaths, emergency department visits related to drug overdose, treatment counts for opioid use disorder, patients receiving prescriptions for buprenorphine, newly diagnosed cases of acute and chronic HCV, and new diagnoses of HIV. For each outcome of interest, yearly, county-level counts are obtained for each of NC’s 100 counties from 2017 to 2021 from the NC Opioid and Substance Use Action Plan (OSUAP) Data Dashboard (Fliss et al. 2023), the 2021 North Carolina Hepatitis B/C Surveillance report (North Carolina HIV/STD/Hepatitis Surveillance Unit 2022), and the 2021 North Carolina HIV Surveillance Report (North Carolina HIV/STD/Hepatitis Surveillance Unit 2021). These data have also been used in (Murphy et al. 2025) to examine spatio-temporal patterns in opioid-related outcomes.

The six outcomes are jointly modeled using a Bayesian hierarchical spatial dynamic one-factor model. Specifically, for each location i, time j, and outcome k, we assume $Y_{\text{ij}}^{\left(k\right)}\sim \text{Poisson}\left(E_{\text{ij}}^{\left(k\right)}\lambda_{\text{ij}}^{\left(k\right)}\right)$, where $\text{log}\left(\lambda_{\text{ij}}^{\left(k\right)}\right)=\gamma_{i}^{\left(k\right)}f_{\text{ij}}+\varepsilon_{\text{ij}}^{\left(k\right)}$ and $\varepsilon_{\text{ij}}^{\left(k\right)}\overset{\text{ind}}{\sim }N\left(0,\sigma_{k}^{2}\right)$. Treatment counts between one and five are censored, which we account for in the model by adapting the censored generalized Poisson regression model of Famoye and Wang (2004). The expected counts, $E_{\text{ij}}^{\left(k\right)}$, are defined using the state average rate in 2017. Specifically, $E_{\text{ij}}^{\left(k\right)}=P_{\text{ij}}r^{\left(k\right)}$, where $P_{\text{ij}}$ is the total population at location i in year j, and $r^{\left(k\right)}$ is the overall empirical state rate of outcome k in 2017. The factors, $f_{\text{ij}}$, are modeled with a dynamic spatial factor model such that

$$f_{i1}\left|f_{-i1}\sim N\left(\mu_{1}+\sum_{l}\frac{w_{\text{il}}}{w_{i+}}\left(f_{l1}-\mu_{1}\right),\frac{\tau_{f}^{2}}{w_{i+}}\right)\right.,\;\text{for}\;j=1$$

$$f_{\text{ij}}\left|\left\{f_{i(j-1)},{\text{f}}_{-\text{ij}}\right\}\sim N\left({\tilde{\mu }}_{\text{ij}}+\sum_{l}\frac{w_{\text{il}}}{w_{i+}}\left(f_{lj}-{\tilde{\mu }}_{lj}\right),\frac{\tau_{f}^{2}}{w_{i+}}\right)\right.,\;\text{for}\;j=2,\ldots ,J.$$

In (13), ${\tilde{\mu }}_{\text{ij}}=\mu_{j}+\eta \left(f_{i(j-1)}-\mu_{j-1}\right)$ and $\mu_{j}$ is an overall intercept for the factor in year j. That is, the factor assumes an intrinsic conditional auto-regressive (ICAR) structure to account for spatial autocorrelation and an autoregressive of order one structure to account for temporal autocorrelation. The $w_{il}$ elements, belong to the neighborhood matrix, which defines the spatial relationships between locations using an adjacency-based approach. That is, two locations are considered neighbors if they share at least one point. Accordingly, the (i,l) element equal to 1 if location i and l are neighbors and 0 otherwise. We let $w_{i+}$ be the total of neighbors for location i.

For model identification, we assume $\gamma_{i}^{\left(1\right)}=1$ for all i, but we allow the loadings, $\gamma_{i}^{\left(k\right)}$ for k=2,…,6, to vary spatially, specifying their structure using an ICAR model with mean one, to reflect the prior assumption that on average, the outcomes are all equally correlated to the latent factor. That is,

(13) $$\gamma_{i}^{\left(k\right)}\left|\gamma_{-i}^{\left(k\right)}\sim N\left(1+\sum_{l}\frac{w_{\text{il}}}{w_{+i}}\left(\gamma_{\text{li}}-1\right),\frac{\tau_{\gamma }^{2}}{w_{+i}}\right).\right.$$

Our model is fit in the Bayesian paradigm, and we assign weakly informative prior distributions for all remaining parameters. The variance components, $\sigma_{k}^{2},\tau_{f}^{2}$, and $\tau_{\gamma }^{2}$ are assumed to have inverse gamma prior distributions with shape and scale parameters of 0.5, and the intercept parameters, $\mu_{j}$, are assumed to have a uniform prior distributions on the real line. We use the nimble package (de Valpine et al. 2017) in R (R Core Team 2021) to implement a metropolis-within-gibbs markov chain monte carlo (MCMC) algorithm to sample from the posterior distribution of all model parameters. The MCMC algorithm is implemented for 1, 000, 000 iterations where the first 500, 000 iterations are discarded as burn-in, and subsequently, every 50th sample is retained. The choice of a longer burn-in and larger thinning interval was motivated by storage considerations. We established convergence by visually inspecting trace plots and by computing the Gelman-Rubin diagnostic. We conducted a sensitivity analysis on the precision parameters by testing alternative prior distributions. In addition to the original Gamma(0.5, 0.5) prior, we considered a Gamma(5, 2) prior. The standard deviation maps of the estimated latent factor showed no meaningful differences between prior specifications. With the model well-identified and relatively non-informative priors, the estimates remained consistent across these prior choices, indicating that our results are not sensitive to prior assumptions (see Figures SM26–SM30 in the supplementary material). The data and code for this analysis are available at https://github.com/evamurphy100/spatialloadings/tree/main.

### 3.2 Opioid syndemic in North Carolina

In this subsection, we explore how the theoretical findings from the previous sections provide a more nuanced understanding of the opioid syndemic in North Carolina. We compare the interaction of six opioid-related outcomes under two scenarios: with spatially varying loadings and with spatially constant (non-varying) loadings.

**Fig. 1:**
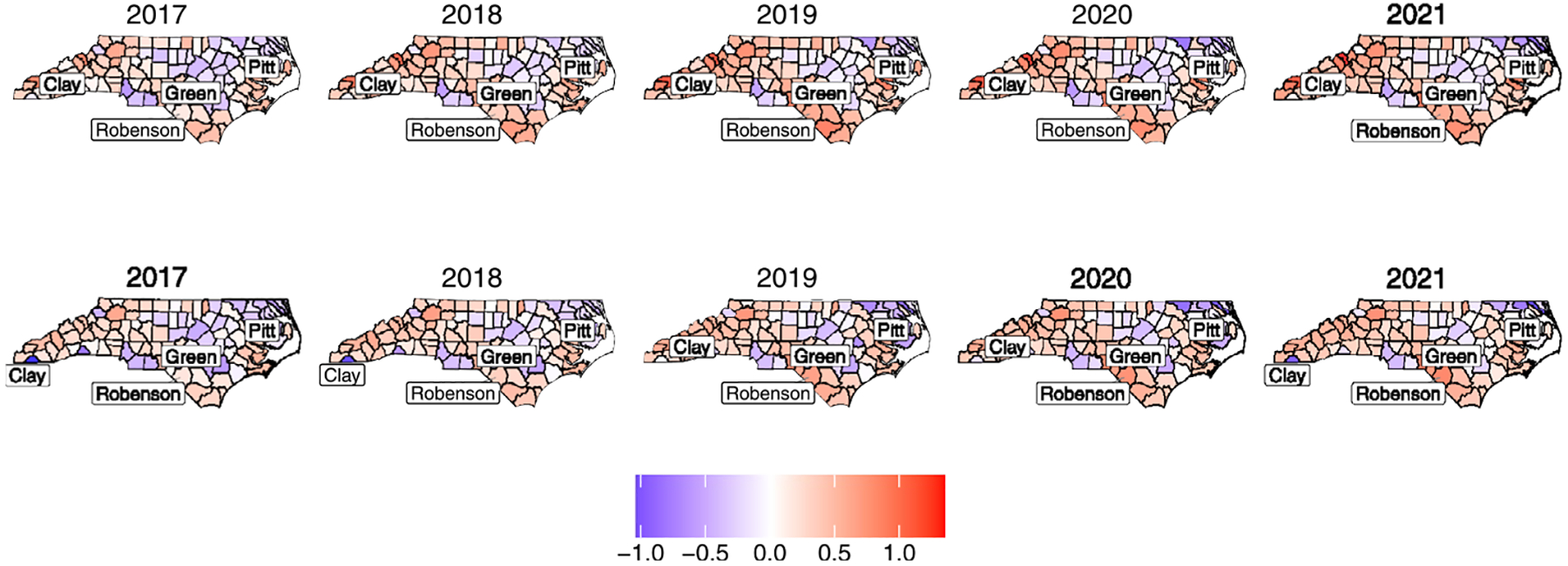
Posterior mean estimates of the latent factor from 2017 to 2021 with constant loadings (top row) and spatially varying loadings (bottom row). Zero indicates the state average in 2017

**Fig. 2:**
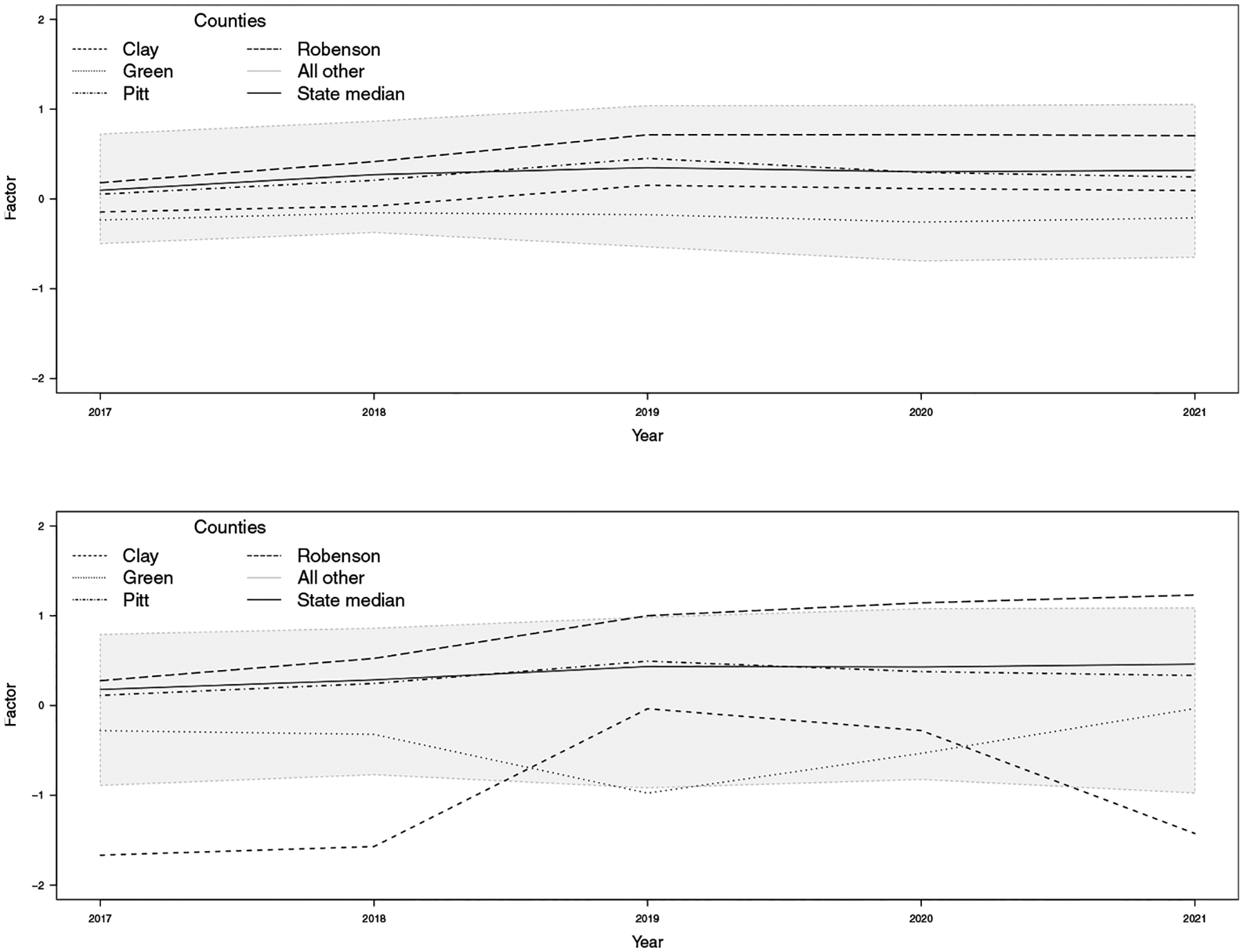
Posterior mean estimates of the latent factor over time with constant loadings (top) and spatially varying loadings (bottom). The grey region represents all counties that fall within the 2.5th quantile and 97.5th quantile of the rates. The state median and specific counties are highlighted using different line types

Maps of the estimated log relative risk for each outcome, shown in Figures SM8–SM13 in the supplementary material for the spatially varying loadings setup and Figures SM14–SM19 for the spatially constant loadings setup, closely resemble the empirical log relative risk maps in Figures SM20–SM25, reassuring the model’s goodness of fit to the data. Figure 1 and 2 compare the posterior means of the estimated latent factor with the two models: one with spatially constant loadings and the other with spatially varying loadings. To illustrate the spatial and temporal variation, we highlighted five counties: Clay County, with a population of 11, 231 people in 2021 as an example for low factor value; Robeson County, with a population of size 130, 625 people in 2021, as an example for high factor value; Pitt County, with a population of size 180, 742 in 2021; and Green County, with a population of size 21, 069 in 2021, as examples for medium factor values. At first glance, both factors appear very similar, showing a relatively subtle increase in the factor.

However, a closer examination of the time series graphs in Fig. 2 reveals prominent differences between the two factors. For example, in Robeson County, starting in 2019, the factor deviates more significantly from the median when loadings vary spatially, compared to when they are constant. In Clay County, the factor with spatially varying loadings shows an evident negative deviation from the median, with a substantial increase between 2018 and 2019 followed by a sharp decrease, in contrast to the factor with constant loadings. In Green County, the factor is significantly below the median when loadings vary spatially, as opposed to when they do not. These differences relate to our conclusion on the covariance structure of the outcome vector, Y. When the loadings are constant, it is assumed that the relationship between the factor (F) and the outcomes (Y) is the same in the spatial domain.

This means that the spatial variation of Y is mainly influenced by the spatial variation of F, forcing F to account for most of the spatial variation in the outcomes across different counties, as previously mentioned. On the other hand, when the loadings vary spatially, they can adjust to how strongly F influences Y in the spatial domain. In this case, the variance of Y depends not only on the variance of F but also on the spatial variability of the loadings, allowing the model to account for regional differences. That is, this additional layer of spatial variation in the loadings can redistribute the observed variance, capturing localized effects. As a result, the trend in the factor may appear pronounced in some counties because spatially varying loadings allow the model to explain the variance of Y differently in each location.

Table 1 shows the posterior mean estimates of the spatially constant loadings. Buprenorphine prescriptions receive the highest weight, with loadings values close to one, as expected, given their strong similarity to treatment counts. Death counts also have a loadings value closer to one, compared to the other outcomes: ED visits, HCV, and HIV, indicating a closer relationship between deaths, buprenorphine prescriptions, and treatment counts in the context of the opioid syndemic. This difference is reasonable, as ED visits include unintentional and undetermined overdoses with the potential for misuse, and HCV and HIV can be transmitted through methods unrelated to opioid use. We note that, under the assumption of spatially constant loadings, the way the factor influences the outcomes—and consequently, how the outcomes relate to the syndemic—remains the same throughout the entire spatial domain of North Carolina. This means that regardless of whether we are in Clay County, Robeson County, Pitt County, or Greene County, the outcomes contribute to the latent opioid syndemic in the same way.

**Fig. 3:**
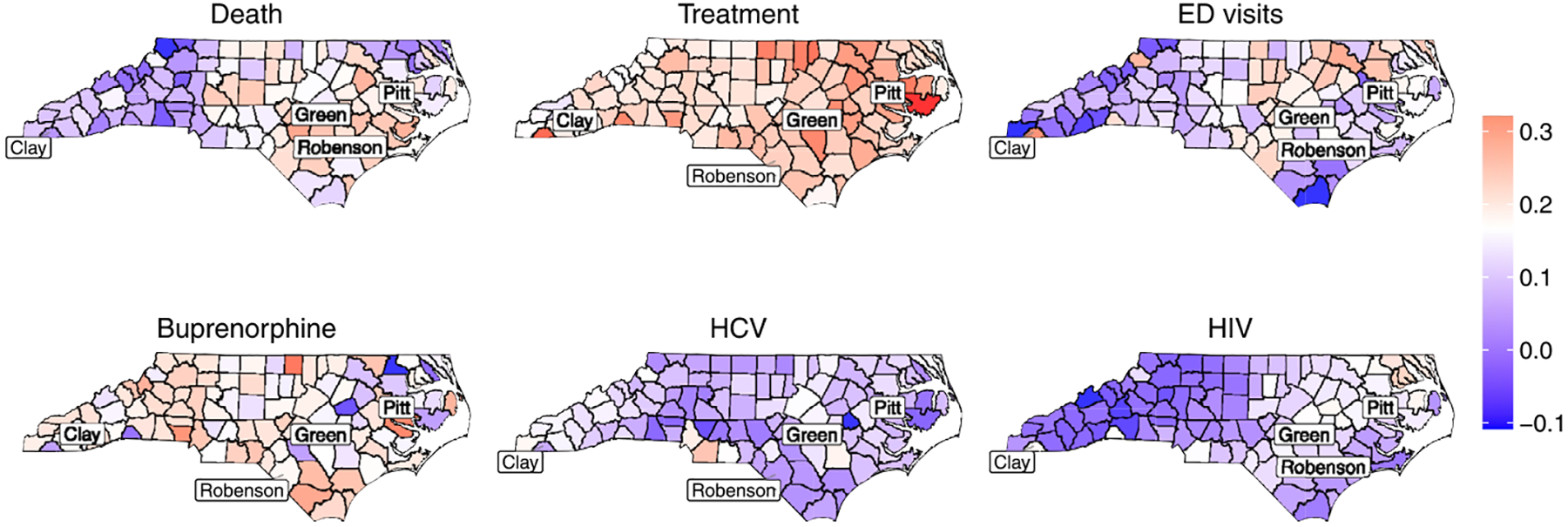
Scaled posterior mean estimates of the spatially varying loadings, computed by dividing each loading by the sum of all six loadings at each location. The color scale is centered at 1/6 (≈ 0.167), where values close to this indicate an equal contribution to the factor. Loadings greater or less than 0.167 represent areas of relatively higher or lower contributions, respectively. First row (left to right): death, treatment, ED visits. Second row (left to right): Buprenorphine, HCV, HIV

With respect to spatially varying loadings, recall that we assume the mean of the loadings of each outcome k,k=2,…,6, is one. Under this assumption, a factor loading of one indicates that an outcome aligns with the latent factor to the same extent as the treatment outcome. The spatial maps of these raw loadings are shown in Figure SM1 of the supplementary material. However, interpreting the raw loadings in this format is challenging, as it requires comparison with the reference outcome, treatment. To facilitate interpretation, we scale the loadings and present the resulting spatial maps in Fig. 3. Scaling the spatially varying loadings involves dividing each loading by the sum of the six outcome loadings at the same location (Neeley et al. 2014). This allows for interpretation relative to 1/6 = 0.167, which serves as a baseline for equal contribution. Since the mean loading is assumed to be one, an outcome with a scaled loading of 0.167 at a given location contributes equally to the latent factor as the other outcomes at that location. Values above 0.167 indicate that the outcome has a stronger agreement with the latent factor, whereas values below 0.167 suggest that the outcome is less associated with the factor. For example, in the case of death counts, western counties such as Clay county have loading values below 0.167, whereas most central and eastern counties, including Robeson, Pitt, and Greene counties, have loadings above 0.167. This suggests that in western counties, death counts have a weaker correlation with the latent factor compared to other outcomes, whereas in central and eastern counties, they play a more prominent role. A similar pattern is observed for ED visits, with lower values in the western counties and higher values in the central-eastern counties. However, an interesting exception is Clay County, where ED visit loadings exceed 0.167, while in Pitt and Greene Counties, they are below this threshold. This suggests that in Clay County, ED visits are more strongly associated with the latent factor, whereas in Pitt and Greene Counties, they are less associated. Loadings for treatment and buprenorphine prescriptions are relatively consistent across North Carolina, with most values above 0.167, suggesting that these outcomes contribute more strongly to the factor. In contrast, loadings for HCV and HIV infections are generally below 0.167 across most counties, indicating that these outcomes are less associated with the latent factor and exhibit more independent behavior. This is expected as HCV and HIV can also be transmitted through non-opioid-related pathways so less variability is shared with the other opioid-related outcomes.

We provide maps of the posterior means of the outcome-specific errors, $\varepsilon^{\left(k\right)},k=1,2,\ldots ,6$, in Figures SM2–SM7 of the supplementary material. These plots suggest that the estimated errors tend to be smaller in the spatially varying loadings scenario compared to the spatially constant loadings scenario.

**Fig. 4:**
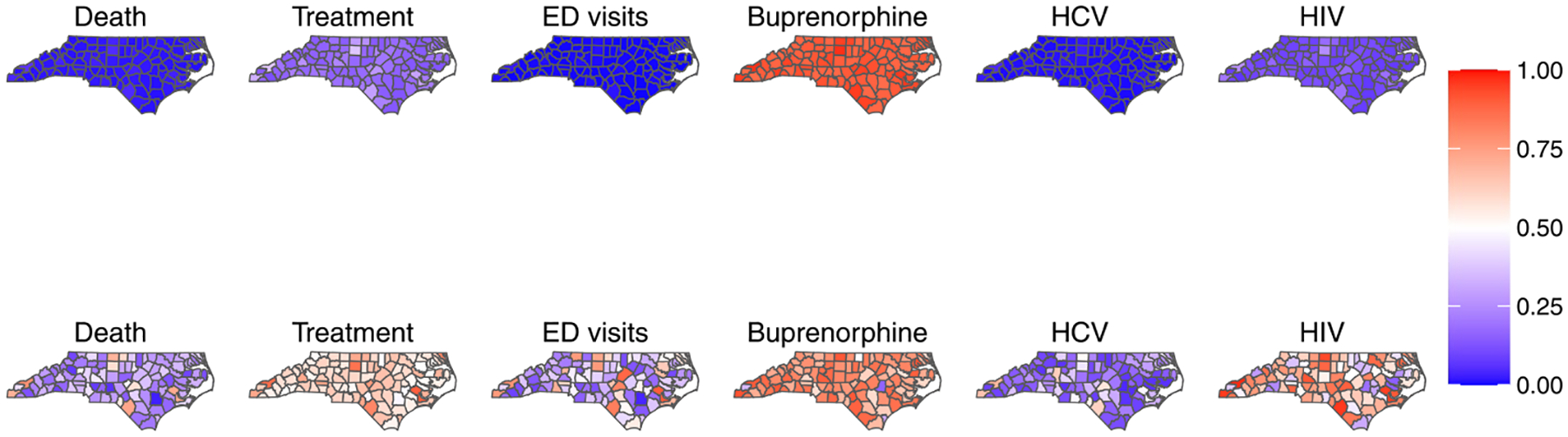
Proportion of variances explained by the product of loadings and factor relative to total variance for each outcome. Top row shows the ratios computed using spatially constant loadings, while the bottom row shows the ratios using spatially varying loadings. Red indicates values above 0.50, while blue represents values below 0.50

To further explore the role of spatially varying loadings, we assess the proportion of variance explained in each outcome. Specifically, we compute the ratio of the variance of the product of the loadings and factor to the total variance in each outcome. Fig. 4 displays these ratios, with red indicating values above 0.50 and blue representing values below 0.50. The figure suggests that ratios for the spatially constant loadings scenario are generally below 0.50, whereas ratios for the spatially varying loadings scenario are more often above 0.50. This indicates that spatially varying loadings account for a larger portion of the total variance compared to the spatially constant loadings. Allowing loadings to vary spatially helps capture more of the local variability in the outcome, whereas the spatially constant loadings provide a more averaged representation.

## 4 Discussion

In this study, we explored the role of spatially varying loadings in a Bayesian hierarchical spatial dynamic one-factor model to better understand the opioid syndemic. The model accounts for the spatial covariance of the outcomes, which consists of three different components: the spatial variability of the loadings, the spatial variability of the factor, and the independent error variance. This decomposition reveals that the spatial variation in an outcome is determined by the combined spatial covariance of the loadings and factors, as well as the additional spatial covariance contribution directly from the factors themselves. The off-diagonal elements of the covariance matrix capture the spatial variation between outcomes, driven by both the shared spatial variability in the loadings and the spatial correlation of the factors. To evaluate the importance of spatially varying loadings, we compared this model with a simpler model where the loadings are spatially constant across locations. When the loadings are constant, the covariance matrix simplifies, and all spatial variation in the outcomes is attributed solely to the spatial distribution of the latent factor.

We applied this model to data from North Carolina, comparing the results from spatially varying loadings to those from a model with spatially constant loadings. Our analysis showed that spatially varying loadings provide more nuanced insights into the dynamics of the opioid syndemic. That is, they allow us to observe critical differences in how outcomes contribute to the syndemic across counties — differences that are not captured under the assumption of spatially constant loadings. For example, in the western part of North Carolina, treatment counts and buprenorphine prescriptions are more related to the latent factor, whereas in the central region, death counts and ED visits also contribute. Spatially constant loadings, by contrast, impose the same structure of interactions between outcomes across the state, overlooking the fact that North Carolina exhibits diverse regional dynamics. Spatially varying loadings allow us to uncover these different structures, providing a clearer picture of how the outcomes interact in different parts of the state. While using spatially varying loadings adds some complexity, it ultimately provides a more informative and localized understanding of the opioid syndemic. By considering county differences in how outcomes interact, we can identify areas where targeted interventions might be most effective. While spatially varying loadings introduce some complexity, they provide a clearer picture of the dynamics at play.

It is important to note a few key aspects of our study. We use factor analysis to quantify the shared structure among six opioid-related outcomes within a confirmatory factor analysis (CFA) framework, where the number of latent factors is prespecified rather than estimated through exploratory methods. We model a single latent factor to align with how the opioid syndemic is defined in public health literature. The opioid syndemic is commonly understood as a set of interconnected and overlapping epidemics—including opioid misuse, overdose, HCV, and HIV—that interact and amplify one another’s effects on a population (Perlman and Jordan 2018; Unick et al. 2013; Valdiserri et al. 2014; Singer et al. 2017). This one-factor structure captures this shared variation while maintaining a simple model that facilitates clear comparison between spatially varying and spatially constant loadings. While beyond the scope of the current study, extending this framework to include multiple factors could be a valuable direction for future research. It is worth clarifying further that our primary motivation for using a fixed 2017 statewide rate to compute expected counts was to provide a baseline reference for interpreting the factor, allowing for consistent comparisons of spatial variation across years. We are not assuming that the expected number of outcomes remains fixed at the 2017 level throughout the study period. Rather, our model assumes the expected value of outcome k at location i and time j is $E_{\text{ij}}^{\left(k\right)}\cdot \lambda_{\text{ij}}^{\left(k\right)}$, where $E_{\text{ij}}^{\left(k\right)}$ is defined using the state average rate in 2017 and λ quantifies differences from this 2017 baseline. The 2017 baseline serves as a standardized denominator that enables meaningful comparisons of relative risk across different years and locations. Finally, our model assumes that spatially dependent factor loadings $\left(\gamma_{i}^{\left(k\right)}\right)$ remain constant over the 2017–2021 study period, which may not reflect the reality that outcomes can change differently across counties over time due to factors such as the pandemic, policy changes, and evolving drug supply patterns. We prioritized understanding spatial variation in loadings before incorporating temporal variation to maintain model interpretability and avoid potential identifiability issues that could arise from loadings varying over both space and time simultaneously.

In conclusion, this study demonstrates the value of incorporating spatial heterogeneity into the loadings of factor models for understanding complex public health phenomena like the opioid syndemic. By allowing loadings to vary spatially, we revealed meaningful regional differences in how opioid-related outcomes interact across North Carolina—insights that would be obscured under the assumption of spatial homogeneity in the loadings. This spatially-informed loadings approach offers a more nuanced framework for identifying where targeted interventions might be most effective, moving toward geographically-tailored public health strategies.

## Supplementary Material

Supplementary Material

**Supplementary Information** The online version contains supplementary material available at https://doi.org/10.1007/s10742-025-00356-7.

## Figures and Tables

**Table 1:** Posterior mean estimates and 95% credible intervals for the spatially constant factor loadings on each outcome

Death	Treatment	ED visits	Buprenorphine	HCV	HIV
0.408 (0.283,0.528)	1	0.184 (0.098, 0.269)	1.234 (1.145, 1.331)	0.236 (0.145,0.329)	−0.932 (−1.104, −0.770)

ED indicates emergency department; HCV, hepatitis C virus; HIV, human immunodeficiency virus

## Data Availability

Data is provided within the manuscript or supplementary information files
