# Supplementary material for: The role of spatially varying loadings in dynamic spatial factor models for modeling the opioid syndemic
Source: Health Serv Outcomes Res Methodol. Author manuscript; Available in PMC 2025 Oct 3. (PMC12490277; doi:10.1007/s10742-025-00356-7)
Supplement: Supplementary Material [file NIHMS2113537-supplement-Supplementary_Material.pdf]

Supplementary material to accompany: The Role of Spatially Varying Loadings in Dynamic Spatial Factor Models for Modeling the Opioid Syndemic

Eva Murphy*, David Kline†, Staci A. Hepler‡

In this supplementary material we present supplementary graphs to our manuscript titled: The Role of Spatially Varying Loadings in Dynamic Spatial Factor Models for Modeling the Opioid Syndemic.

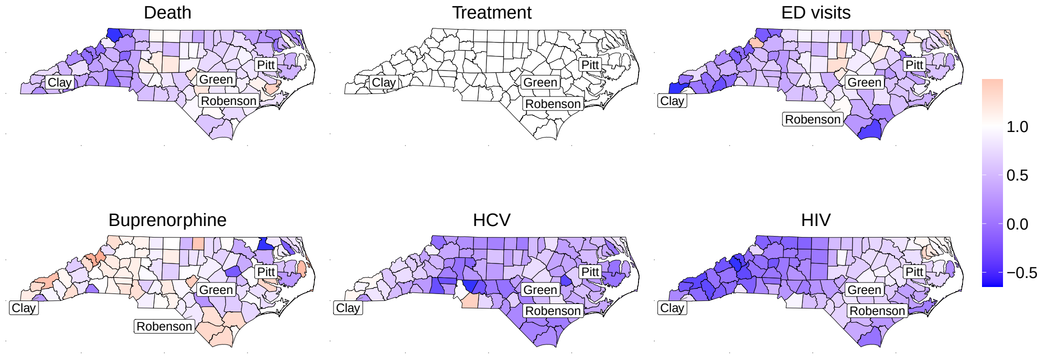

Figure SM1: Posterior mean estimates of the raw, not centered, spatially varying loadings.

*Wake Forest University. E-mail: murphye@wfud.edu
†Wake Forest University School of Medicine. E-mail: dkline@wakehealth.edu
‡Wake Forest University. E-mail: heplersa@wfu.edu

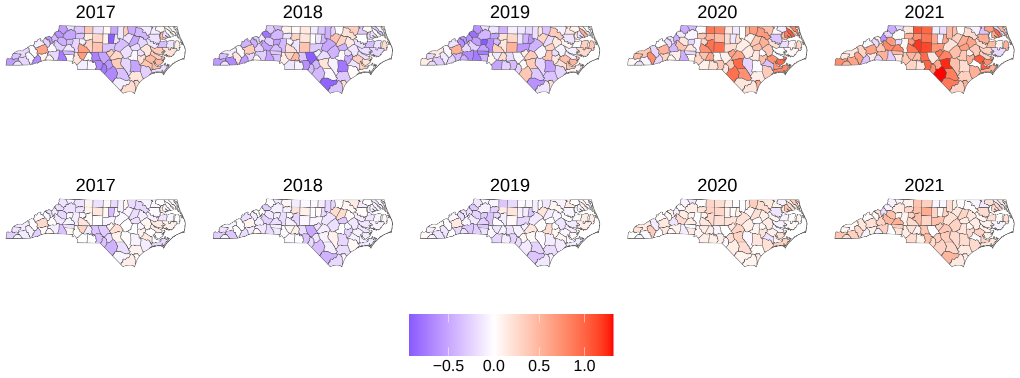

Figure SM2: Posterior mean estimates of the $\varepsilon^{(D)}$ with spatially constant loadings (**top row**) and with spatially varying loadings (**bottom row**).

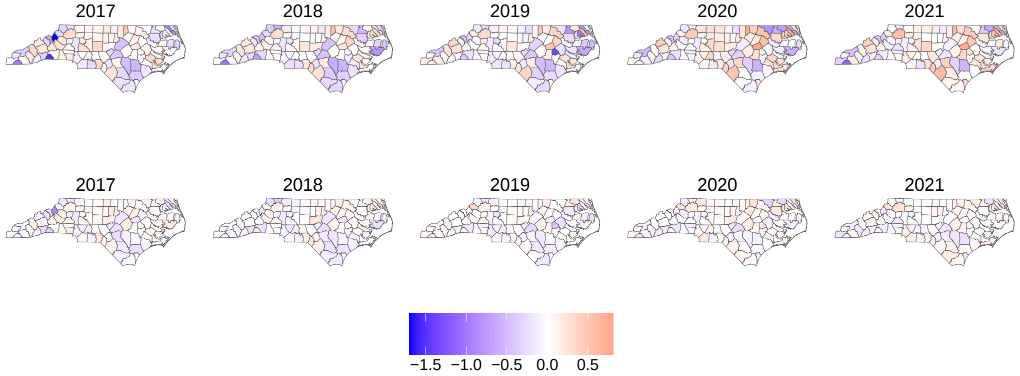

Figure SM3: Posterior mean estimates of the $\varepsilon^{(T)}$ with spatially constant loadings (**top row**) and with spatially varying loadings (**bottom row**).

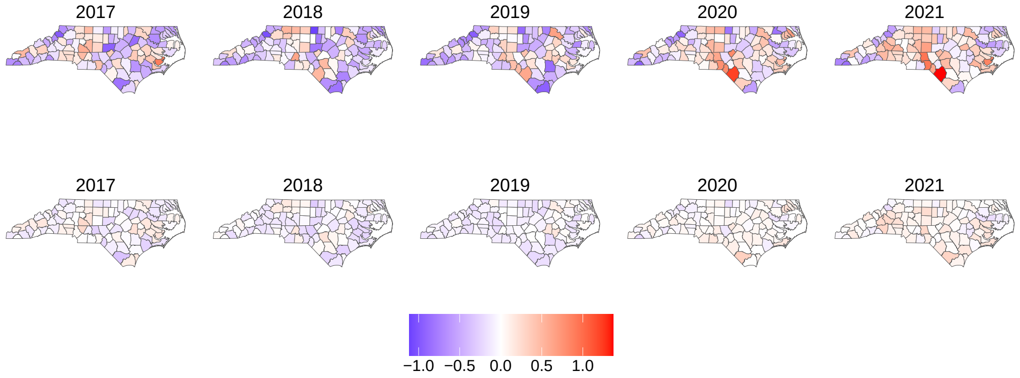

Figure SM4: Posterior mean estimates of the $\varepsilon^{(E)}$ with spatially constant loadings (**top row**) and with spatially varying loadings (**bottom row**).

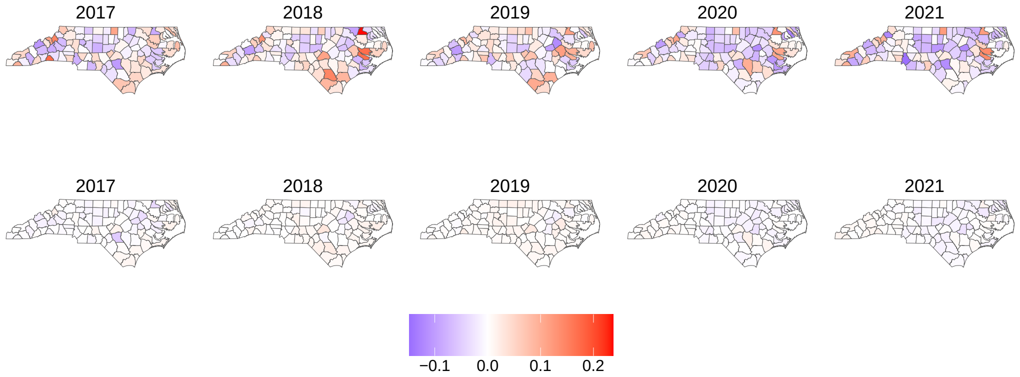

Figure SM5: Posterior mean estimates of the $\varepsilon^{(B)}$ with spatially constant loadings (**top row**) and with spatially varying loadings (**bottom row**).

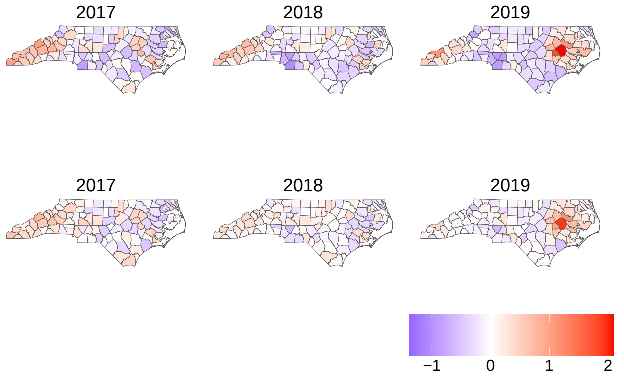

Figure SM6: Posterior mean estimates of the $\varepsilon^{(C)}$ with spatially constant loadings (**top row**) and with spatially varying loadings (**bottom row**).

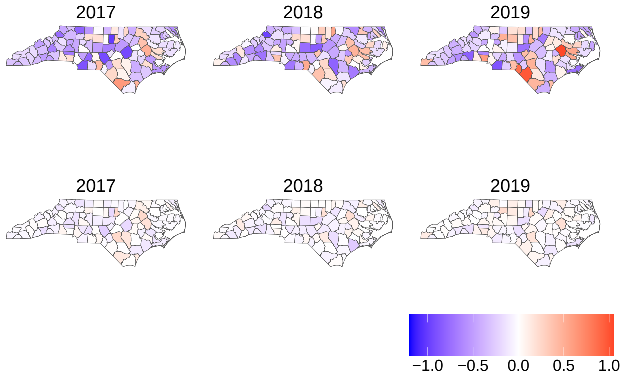

Figure SM7: Posterior mean estimates of the $\varepsilon^{(I)}$ with spatially constant loadings (**top row**) and with spatially varying loadings (**bottom row**).

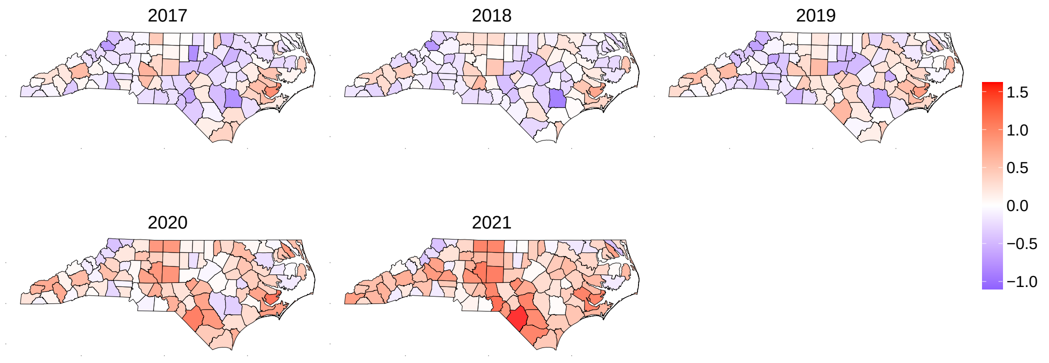

Figure SM8: Posterior mean estimates of the log relative risk death counts with spatially varying loadings.

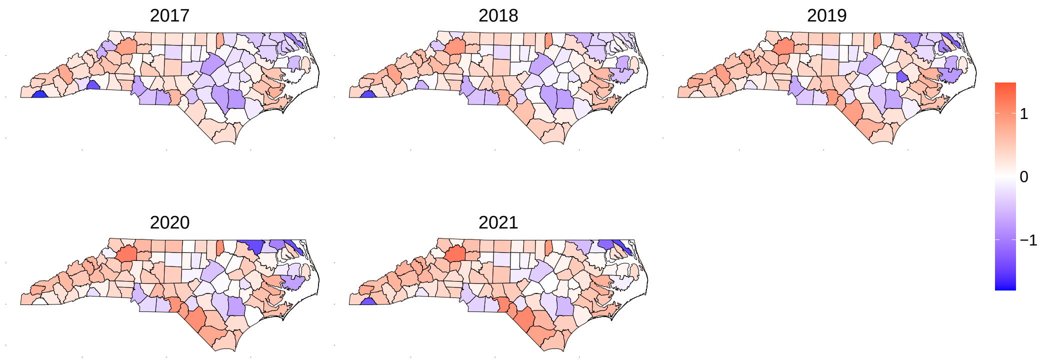

Figure SM9: Posterior mean estimates of the log relative risk of treatment counts with spatially varying loadings.

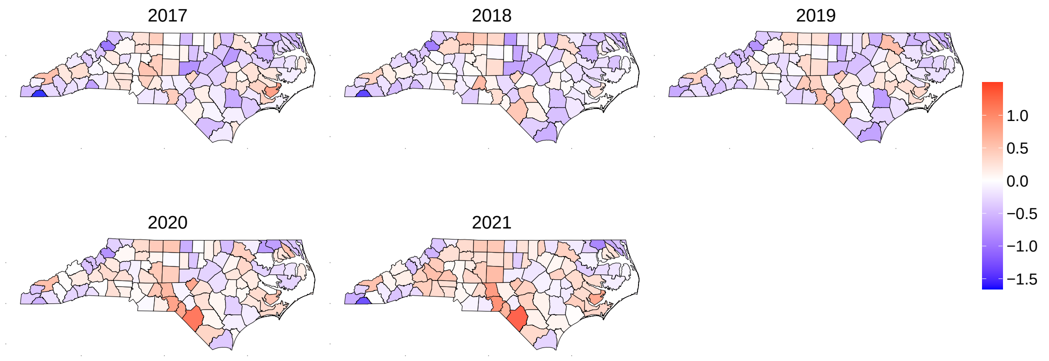

Figure SM10: Posterior mean estimates of the log relative risk of emergency visit counts.

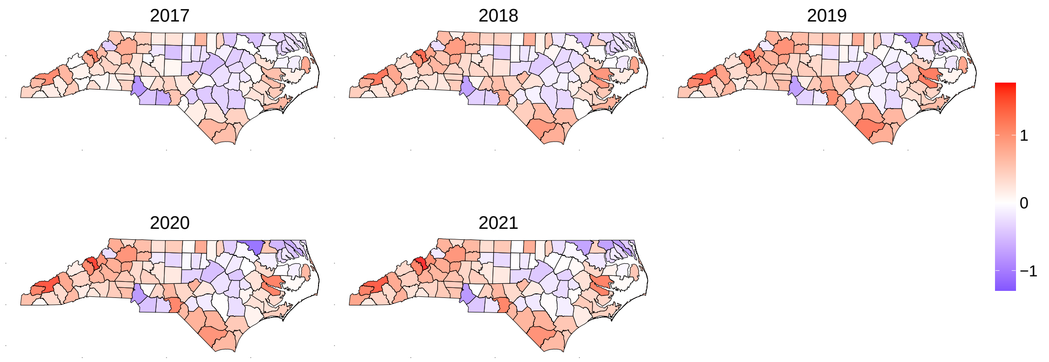

Figure SM11: Posterior mean estimates of the log relative risk of buprenorphine prescription counts with spatially varying loadings.

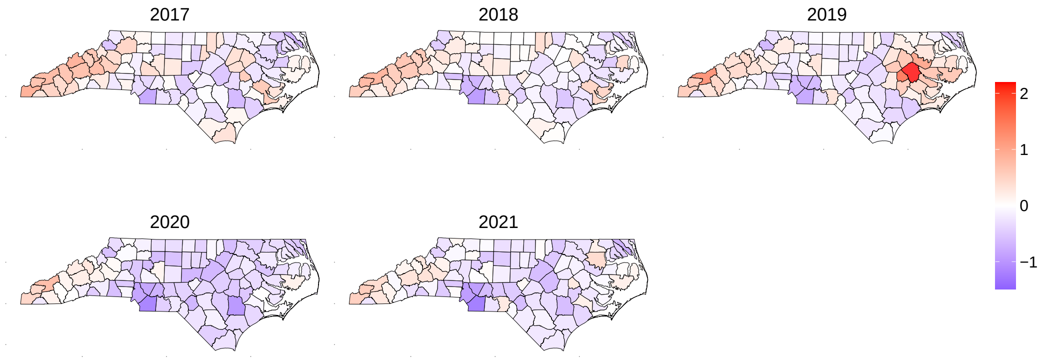

Figure SM12: Posterior mean estimates of the log relative risk of HCV infection counts with spatially varying loadings.

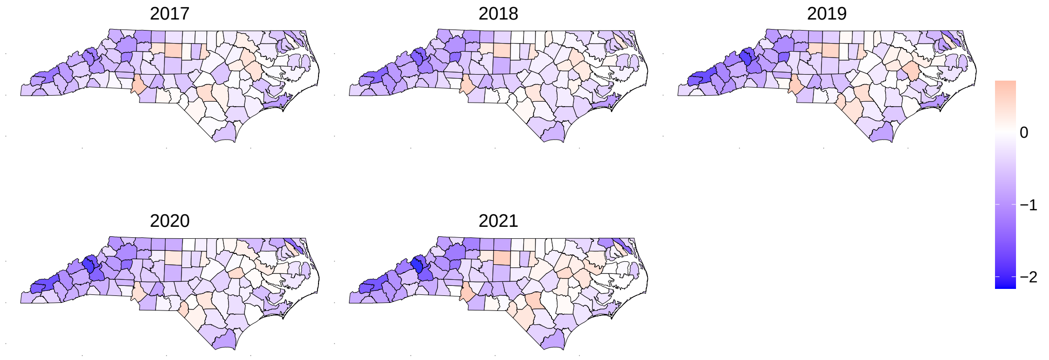

Figure SM13: Posterior mean estimates of the log relative risk of HIV infection counts with spatially varying loadings.

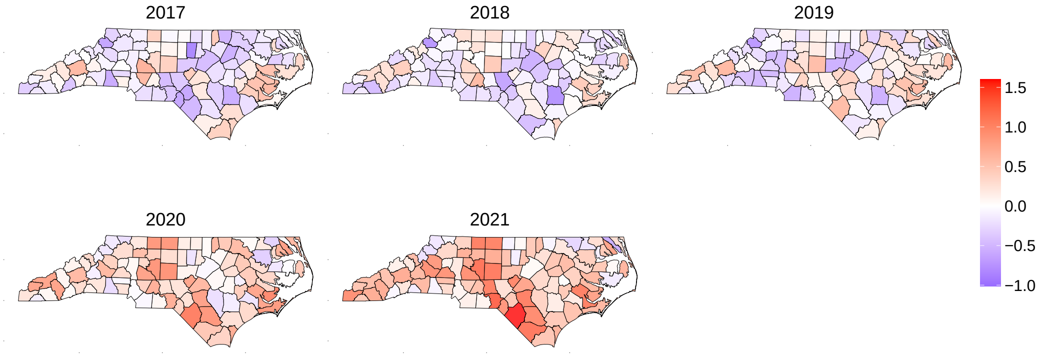

Figure SM14: Posterior mean estimates of the log relative risk death counts with spatially constant loadings.

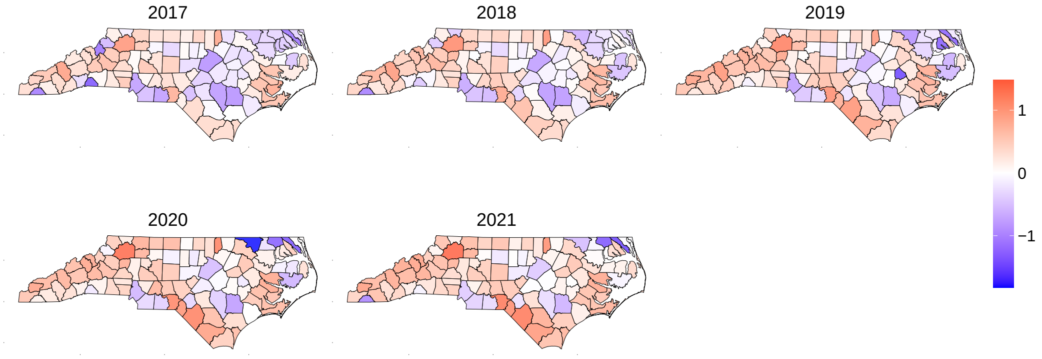

Figure SM15: Posterior mean estimates of the log relative risk of treatment counts with spatially constant loadings.

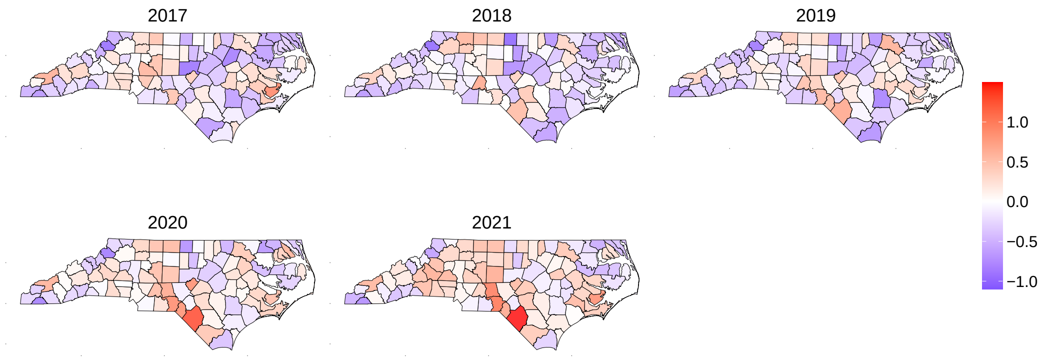

Figure SM16: Posterior mean estimates of the log relative risk of emergency visit counts with spatially constant loadings.

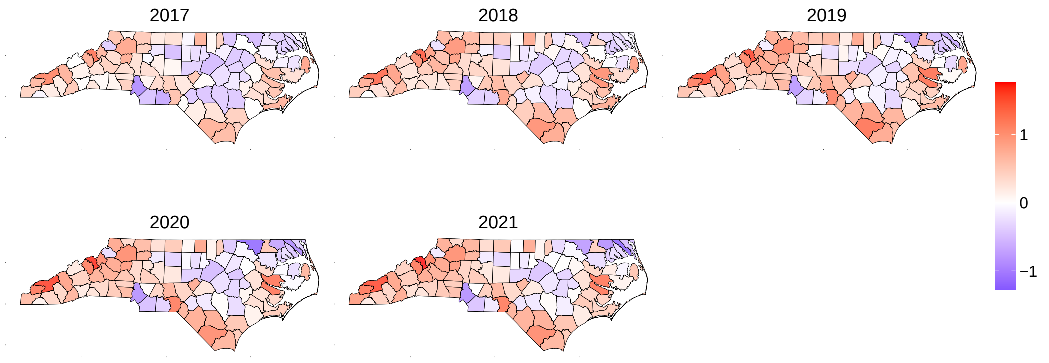

Figure SM17: Posterior mean estimates of the log relative risk of buprenorphine prescription counts with spatially constant loadings.

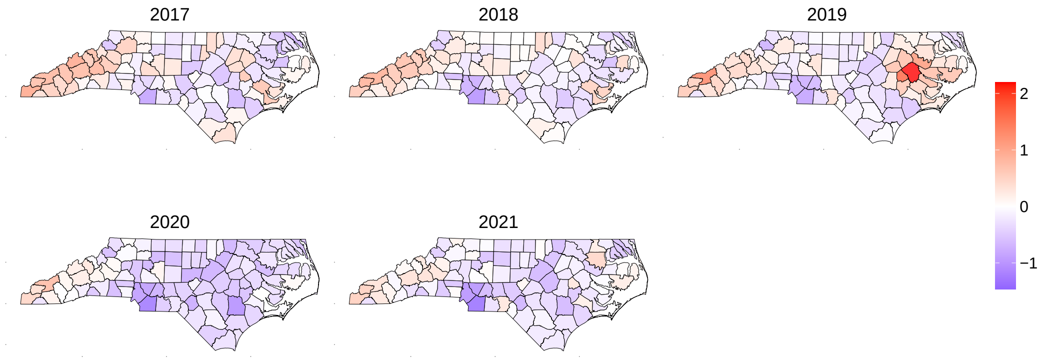

Figure SM18: Posterior mean estimates of the log relative risk of HCV infection counts with spatially constant loadings.

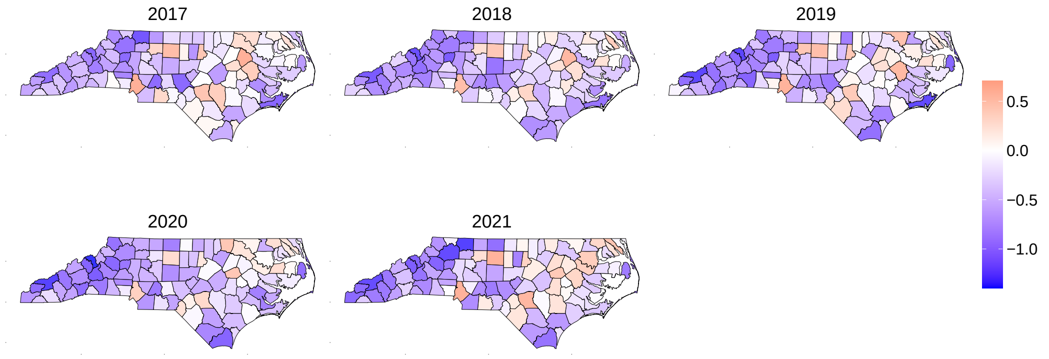

Figure SM19: Posterior mean estimates of the log relative risk of HIV infection counts with spatially constant loadings.

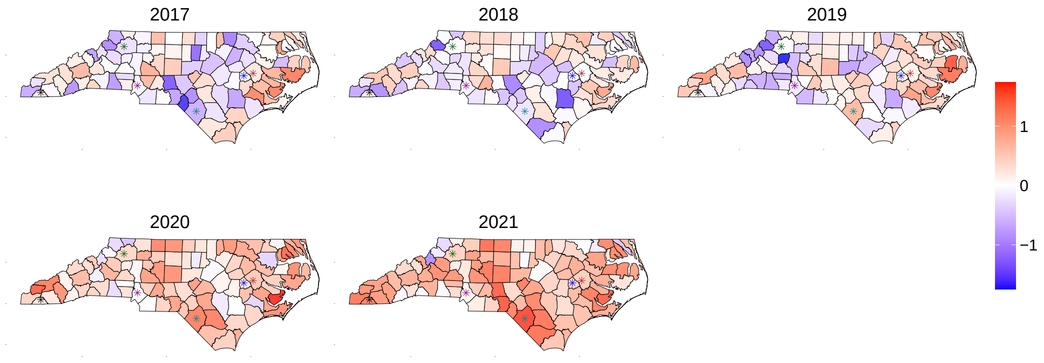

Figure SM20: Observed log standardized relative risk for death counts.

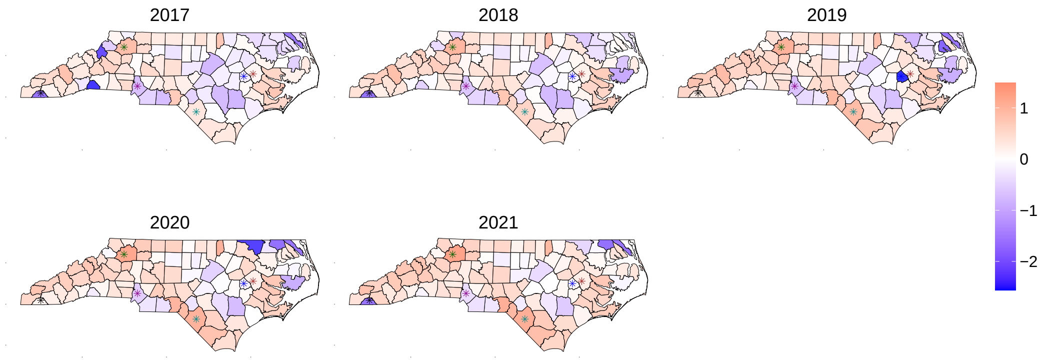

Figure SM21: Observed log standardized relative risk for treatment counts

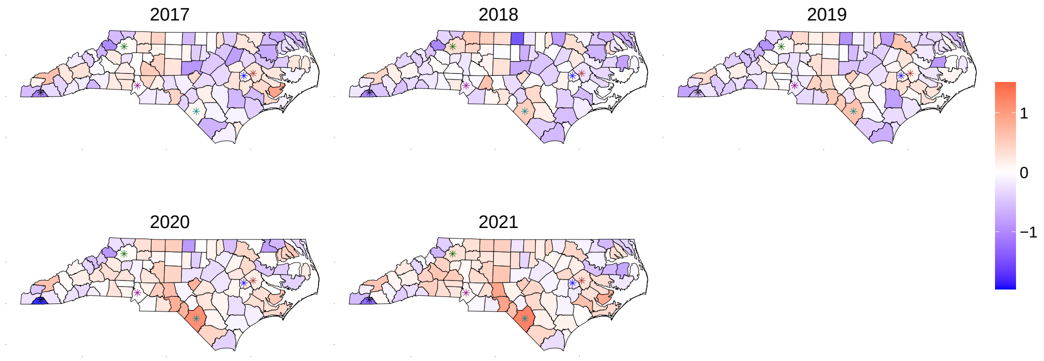

Figure SM22: Observed log standardized relative risk for E.D. visits.

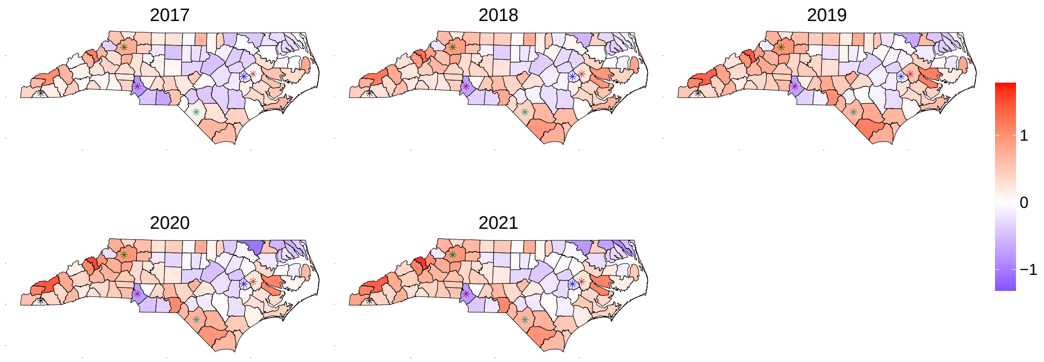

Figure SM23: Observed log standardized relative risk for buprenorphine prescriptions.

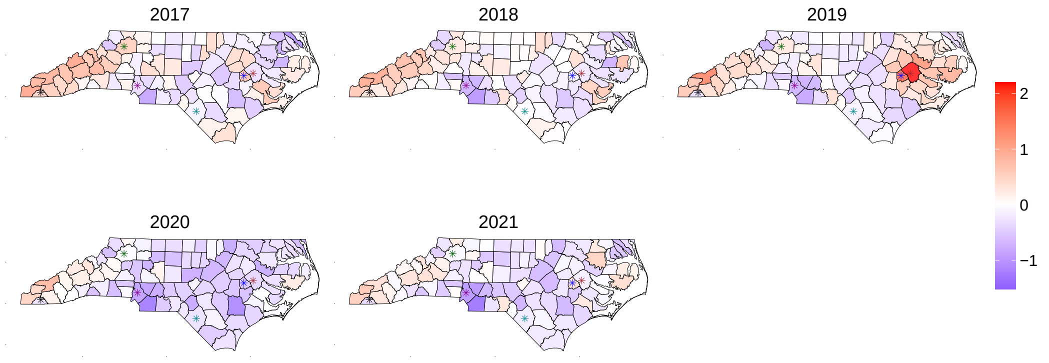

Figure SM24: Observed log standardized relative risk for HCV infection counts.

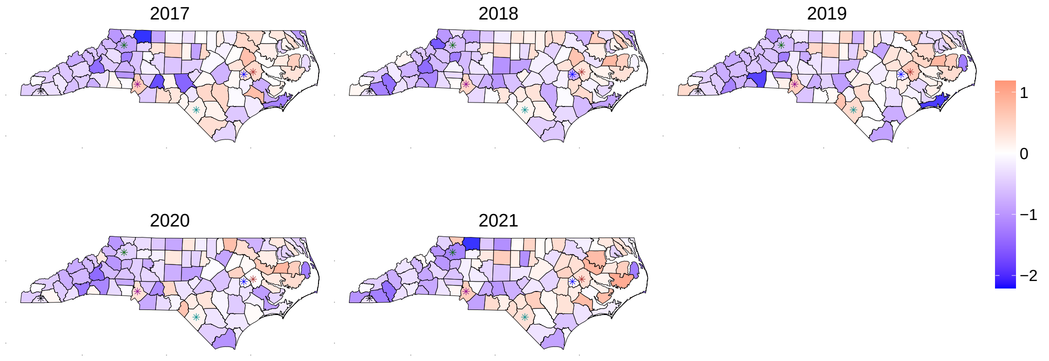

Figure SM25: Observed log standardized relative risk for HIV infection counts.

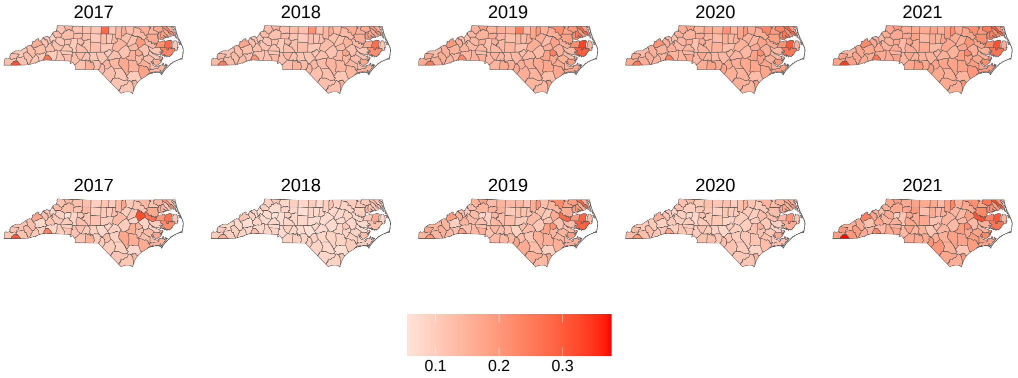

Figure SM26: Posterior standard deviation estimates of the latent factor under Gamma(5,2) (**top row**) and Gamma(0.5, 0.5) (**bottom row**) prior distributions for the precision parameters.

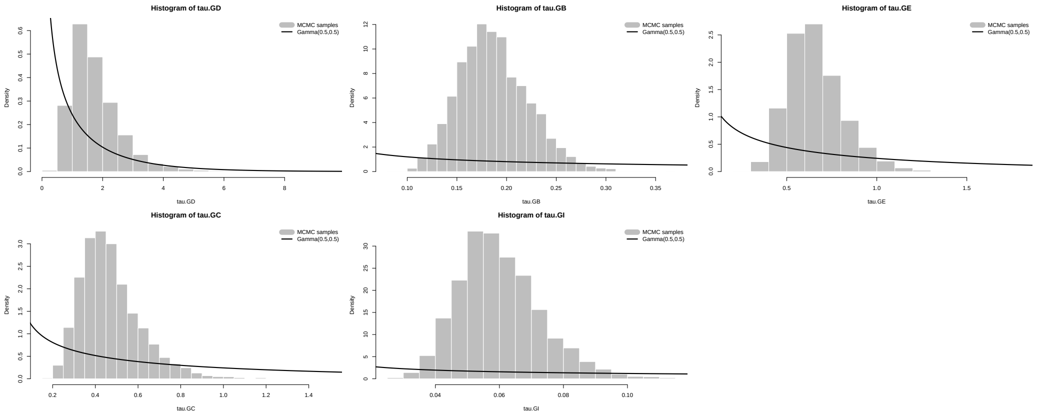

Figure SM27: Posterior distributions of precision parameters for the loadings (tau.GD: precision for loadings pertaining to D; tau.GB: precision for loadings pertaining to B; tau.GE: precision for loadings pertaining to E; tau.GC: precision for loadings pertaining to C; tau.GI: precision for loadings pertaining to I, where D, E, B, C, I are defined in the main text) with Gamma(0.5, 0.5) prior densities overlaid (**black curves**).

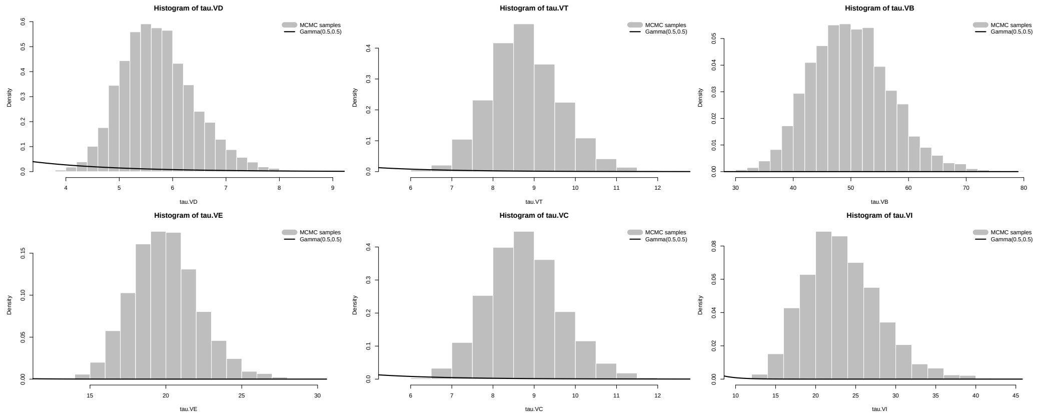

Figure SM28: Posterior distributions of precision parameters for the $\varepsilon^{(k)}$ (tau.VD: precision pertaining to $\varepsilon^{(D)}$; tau.VT: precision pertaining to $\varepsilon^{(T)}$; tau.VB: precision pertaining to $\varepsilon^{(B)}$; tau.VE: precision pertaining to $\varepsilon^{(E)}$; tau.GC: precision pertaining to $\varepsilon^{(C)}$; tau.GI: precision pertaining to $\varepsilon^{(I)}$, where D, T, E, B, C, I are defined in the main text) with Gamma(0.5, 0.5) prior densities overlaid (**black curves**).

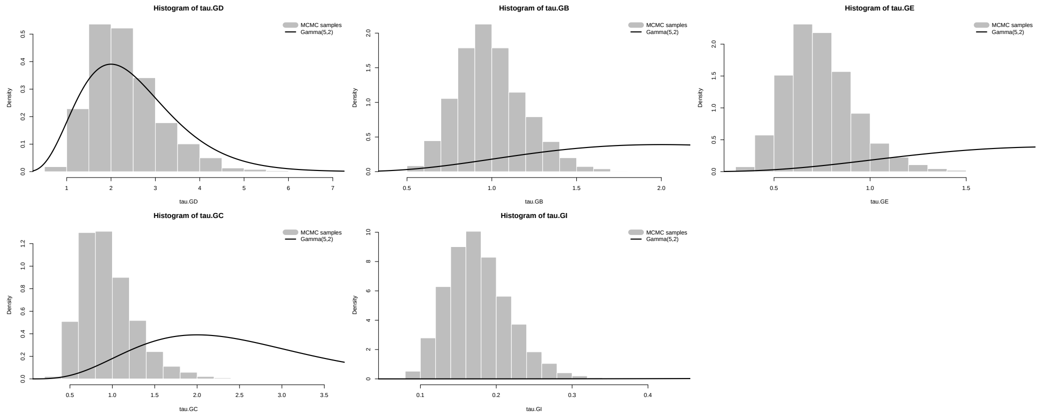

Figure SM29: Posterior distributions of precision parameters for the loadings (tau.GD: precision for loadings pertaining to D; tau.GB: precision for loadings pertaining to B; tau.GE: precision for loadings pertaining to E; tau.GC: precision for loadings pertaining to C; tau.GI: precision for loadings pertaining to I, where D, E, B, C, I are defined in the main text) with Gamma(5, 2) prior densities overlaid (**black curves**).

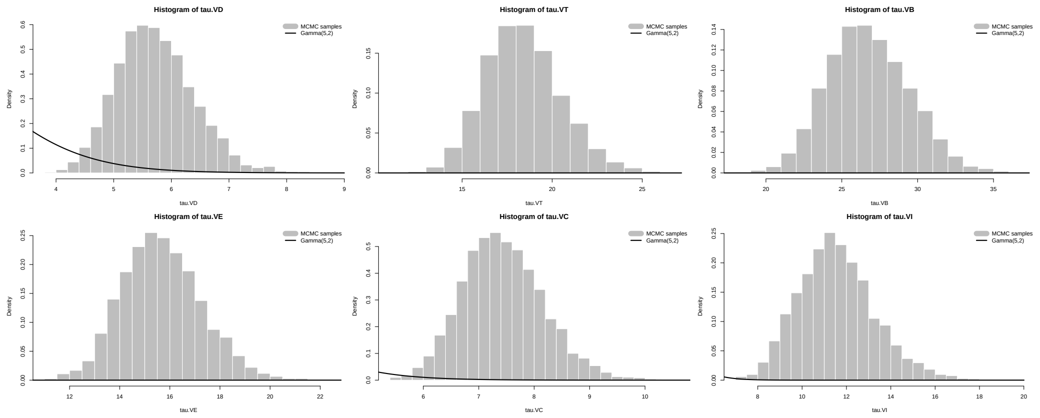

Figure SM30: Posterior distributions of precision parameters for the $\varepsilon^{(k)}$ (tau.VD: precision pertaining to $\varepsilon^{(D)}$; tau.VT: precision pertaining to $\varepsilon^{(T)}$; tau.VB: precision pertaining to $\varepsilon^{(B)}$; tau.VE: precision pertaining to $\varepsilon^{(E)}$; tau.GC: precision pertaining to $\varepsilon^{(C)}$; tau.GI: precision pertaining to $\varepsilon^{(I)}$, where D, T, E, B, C, I are defined in the main text) with Gamma(5, 2) prior densities overlaid (**black curves**).
